# Changes in volumetric bone mineral density after ACL reconstruction with single-bundle and double-bundle: a 1-year follow-up study using peripheral quantitative computed tomography

**DOI:** 10.3389/fbioe.2025.1606404

**Published:** 2025-05-22

**Authors:** Binbin Yin, Chaohua Fang, Ren hai Feng, Jian min Wu

**Affiliations:** ^1^ Department of Radiology, Ningbo No. 6 Hospital, Ningbo, Zhejiang, China; ^2^ Department of Sports Medicine, Shanghai General Hospital, Shanghai Jiao Tong University School of Medicine, Shanghai, China

**Keywords:** ACL reconstruction, tibia, single-bundle, double-bundle, bone mineral density

## Abstract

**Background:**

The purpose of this study is to quantify changes in volumetric bone mineral density (vBMD) in different regions of the tibial plateau after single-bundle (SB) and double-bundle (DB) anterior cruciate ligament reconstruction (ACLR).

**Methods:**

Twenty-six patients with diagnosed anterior cruciate ligament (ACL) rupture were allocated into SB (10) or DB group (16) and completed the last follow-up at 12 months. Peripheral quantitative computed tomography (pQCT) was performed before surgery and at 1, 3, 6, and 12 months after surgery. Four regions of interest (ROI) were identified 2 mm below the medial and lateral subchondral plate of the knee joint, namely, the anteromedial (AM), posteromedial (PM), anterolateral (AL) and posterolateral (PL) regions. The vBMD of each ROI was measured and compared between the groups at different timepoints.

**Results:**

For the vBMD, a significant change in all ROIs can be found over time, with the values at all ROIs decreased until 6 months postoperatively and then steadily increased, but the values did not reach preoperative levels. The AM region had the highest vBMD, followed by the PL region, and the vBMD was lowest in the AL region. For the change percent, the decrease magnitude was comparable between AM and PM region at postoperative 1 and 12 months, but it was greater at PM region at postoperative 3 and 6 months. Comparing DB ACLR and SB ACLR, there was no significant difference in the change percent of vBMD in all ROIs, except for in the AL region at 1 month postoperatively.

**Conclusion:**

A partially reversible decline in vBMD was found in the proximal tibia at 12 months after ACLR. But the change percent varied among the different regions, which may indicate inadequate restoration of knee kinematics after ACLR. DB and SB ACLR methods may have a similar effect on knee kinematics.

## Background

Tearing of the anterior cruciate ligament (ACL) is one of the most common ligament injuries of the knee. In order to counteract excessive anterior-posterior tibial translation and rotational laxities result from such injuries, anterior cruciate ligament reconstruction (ACLR) is often needed (Grassi et al., 2019). Traditionally, Single-Bundle (SB) ACLR techniques are the standard surgical approach and are targeted to recreate the function of the anteromedial (AM) bundle. But recent *in vivo* studies have demonstrated inadequate restoration of rotational stability during daily life activities when using SB ACLR, which led to the development of the double-bundle (DB) concept and reconstruction technique, aimed at better reproducing the anatomical insertion and physiological ACL behavior (Hemmerich et al., 2011; Kim et al., 2015).

No matter which reconstruction technique is used, it is considered that the bone mineral density (BMD) in the proximal tibia of the ipsilateral leg is significantly affected by ACLR and subsequent immobilization (Birch et al., 2018). And low bone mineral density has been associated with the onset of spontaneous osteonecrosis of the knee (Akamatsu et al., 2012). However, how the BMD changes over time is still controversial, including the magnitude of bone loss and time to recovery (Lui et al., 2012; Mundermann et al., 2015; Zerahn et al., 2006). It is also unclear how SB or DB ACLR affect BMD and which method can better restore the BMD to a near-physiological level. Differing outcomes from studies on BMD after ACLR may partly be explained by the different methods used to detect BMD, such as dual-photon absorptiometry, dual-energy X-ray absorptiometry and peripheral quantitative computed tomography (pQCT) (Lui et al., 2012; Mundermann et al., 2015; Zerahn et al., 2006). pQCT can not only capture the change of bone mineral content but also the microstructure of trabecular and cortical bone. Taking advantage of its three-dimensional images, it is possible to measure the bone mineral content in a specific volumetric area of the tibial plateau.

The purpose of this study was to quantify the volumetric bone mineral density (vBMD) in the specific regions at ipsilateral tibial plateau pre- and post DB or SB ACLR. And changes of vBMD were analysed according to region, timepoint, and type of ACLR. It was hypothesized that vBMD decreases in all regions of the tibial plateau immediately after ACLR, but then recovers with varying degrees in the different regions by 12 months, which reflect the kinematics of the knee. And the recovery would be different between DB and SB ACL reconstruction.

## Methods

This study is a Level III, therapeutic, cross-sectional study involving patients undergoing ACLR, which had been approved by the local Institutional Review Board (approval number: K2021067). The inclusion criteria of recruited patients (i) who were suffered from acute trauma within 6 weeks, (ii) who had symptoms of knee instability and other clinical evidence of ACL insufficiency verified by positive Lachman tests, (iii) whose MRI scans revealed no other ligament injury except for ACL rupture, with or without accompanied meniscus injury, (iv) who had not undergone previous ipsilateral knee joint surgery, and (vi) who had not been treated with medications known to affect bone metabolism. The patients were treated with arthroscopic SB ACLR (Group SB) or DB ACLR (Group DB). They were allocated to either group by the performing surgeon considering their age, daily activity level and size of the femur condyle. Insufficiencies in knee ligaments, except for the ACL, were tested and excluded under anesthesia before surgery. A standard diagnostic arthroscopic procedure was performed to verify complete rupture of the ACL. Any accompanying meniscal tears were treated with menisectomy or repair before ACL reconstruction. Standard surgical equipment was used to perform all ACL surgical reconstructions. Group SB underwent a single-bundle ACL reconstruction using semitendinosus and gracilis tendons autografts plus a musculus tibialis anterior allograft, as reported by Pearle AD et al., with an I.D.E.A.L. (Isometric, Direct fibers, Equidistant and Eccentric, Anatomic, Low in tension) femoral tunnel position and tibial ACL footprint (Pearle et al., 2015). Patients in Group DB underwent an anatomic double-bundle reconstruction using semitendinosus and gracilis tendons autografts plus two musculus tibialis anterior allografts (Chhabra et al., 2006). All grafts were fixed by press-fitting with a PEEK interference screw (Smith & Nephew, Memphis, TN, United States) in the tibial tunnels and suspended with an EndoButton (Smith & Nephew Endoscopy, Mansfield, Massachusetts) on femoral side. In order to exclude the effect of the operation on the value of vBMD, pQCT was performed at 1 day postoperatively and the value was deemed as the preoperative vBMD.

Within 2 weeks after surgery patients were encouraged to perform flexibility exercises, namely, ankle pumps, straight-leg-raising movements and ambulating with a brace locked straight. Walking with crutches without weight bearing on the ipsilateral leg was also encouraged. Once the patient could perform some of these basic movements, the brace was unlocked and the knee was allowed flex and the flexion angle was gradually increased over the coming weeks. At 2 months postoperatively, partial weight bearing was allowed and gradually moved to full weight bearing at 3 months postoperatively (Jiang et al., 2011). Daily activities were permissible but strenuous physical exertion and sports activities were avoided until at least 6 months after surgery. Patients were encouraged to return to moderate physical work and simple sports activities such as jogging after 1 year. If rehabilitation was satisfactory and there were no signs of impairment, patients were allowed to return to activities performed at a preinjury level, such as sport and exercise.

**FIGURE 1:**
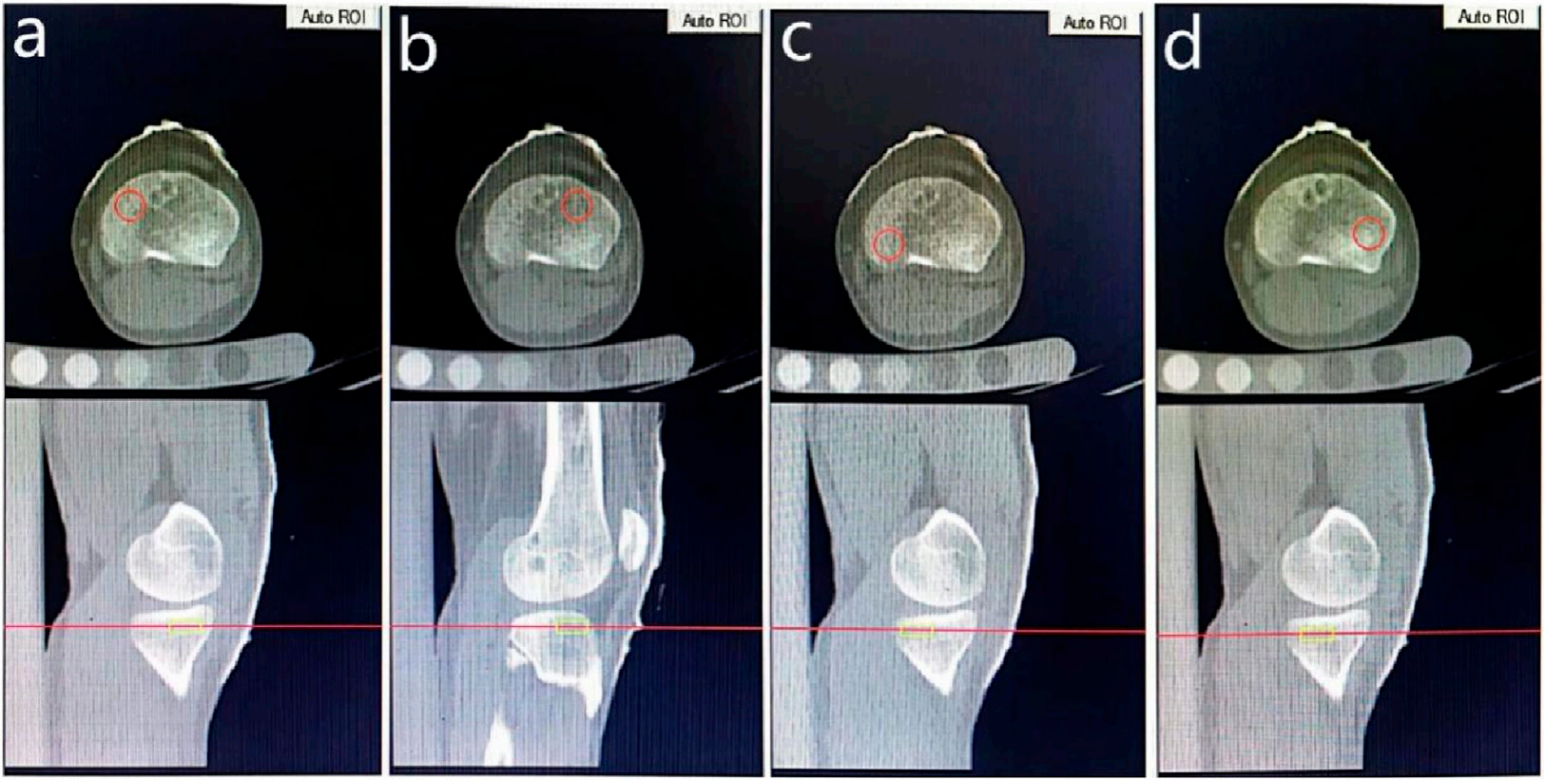
The location and boundary of ROIs, with **(a–d)** indicating the anteromedial, anterolateral, posteromedial and posterolateral areas respectively.

All patients were followed up at 1, 3, 6, and 12 months after surgery. Clinical assessments were performed in the outpatient department, including an assessment of the joint range of motion, thigh circumference, drawer test, Lachman test, and pivot-shift test. Rehabilitation exercises were encouraged for patients with a limited range of motion (ROM) or obvious loss in thigh circumference. The patients with obviously limited ROM or residual drawer test(+), Lachman test(+), pivot-shift test(+) were excluded at follow up. pQCT was performed using Siemens Definition AS 64-row CT machine (Gammatec, Vaerloese, Denmark) at each timepoint, after informed consent was obtained from each participant. The pQCT scanning process required a phantom to be placed underneath the ipsilateral knee, with the volunteers in a supine position and both knees fixed in a neutral position using a frame while maintaining full extension. The scans ranged from 10 cm proximal to 10 cm distal to the joint line with a layer thickness of 1 mm and were taken at a voltage of 120 kV, current of 250 mAs, pitch of 0.5, scanning time of 9.95 s, and field of view of size 500 mm × 500 mm. The images of the knee and phantom were imported into the software workstation (Mindways Software Inc. of the United States) for analysis. Patients showing obvious enlargement of the femoral or tibial tunnel on CT images were excluded in the subsequent follow up. vBMD measurements for cylindrical regions of interest (ROI) were performed on the proximal tibia of the operated knee joint. Firstly, Sagittal images were referenced for determining the axial image: 2 mm below the medial and lateral subchondral plate of the knee joint. Secondly, four ROIs were identified at the axial images, namely, the anteromedial (AM) region, posteromedial (PM) region, anterolateral (AL) region and posterolateral (PL) region: A round or oval shaped circle was set as the boundary of each ROI but exclude the cortical bone. All ROIs were assigned a depth of 7 mm. The size of circle was individually adjusted according to the size of the tibial cross-section, but kept constant for each ROIs and scans at different timepoints of the same patient (Figure 1). In the workstation, the module for Volumetric Spine was used and the value of vBMD was calculated by calibrating the Hounsfield unit of each ROI on the CT images with the hydroxyapatite in the phantom. In this way, the mean value of vBMD for a cylindrical ROI in the subchondral bone could be measured. Because of the press-fit technique, axial images showed the interference screw as one or two cylinders, which was not included when calculating the vBMD by individually adjusting the location of ROI. Two observers recorded vBMD value of each ROI at each timepoint, intra- and interobserver reliabilities were assessed by intraclass correlation coefficients (ICC (1,1) and ICC (2,1), respectively).

### Statistical methods

To minimize individual bias in calculating the vBMD and to quantify changes in vBMD over time, the postoperative vBMD at each timepoint was transformed to the “change percent of vBMD,” calculated as (postoperative vBMD - preoperative vBMD)/preoperative vBMD*100. Differences in vBMD values between the four regions at the same timepoint, or between five timepoints for the same region, were compared using a compatibility group design analysis of variance and SNK-q test between groups. Wilcoxon matched-pairs tests were used to compare the change percent of vBMD between AM and PM regions or AL and PL regions at the same timepoint. Comparisons between the subgroups DB ACLR and SB ACLR were performed using a Mann Whitney U test. *p* < 0.05 was considered statistically significant. Post-hoc analyses were conducted to verify the true powers achieved by the current sample size for supporting the main conclusions (with alpha = 0.05). Analysis of variance was performed using a general linear model to calculate ICC. ICC (1,1) was calculated from data derived from two measurements performed by one observer. ICC (2,1) was determined from the average of the measurements of each of the two independent observers, who were both well-trained radiologists. An ICC >0.7 was considered as almost perfect reproducibility (Landis and Koch, 1977).

## Results

### Patients

**FIGURE 2:**
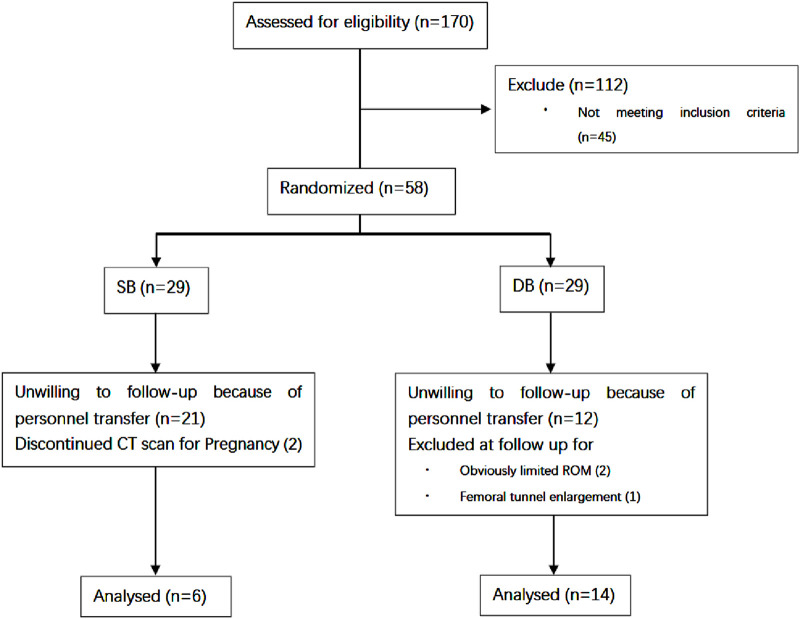
A flowchart showing the flow of patients in the study.

Of the 170 patients undergoing ACL reconstruction at our center between April 2017 and April 2020, 58 patients met the inclusion criteria and were willing to receive postoperative CT scans. Five patient was excluded at 6 months due to obviously limited ROM, femoral tunnel enlargement or pregnancy. A total of 20 patients (4 females and 16 males) completed the last follow-up at 12 months (Figure 2). The average age at the time of surgery was 32.7 years (SD 9.3 years; range 21–53 years). Time from injury to reconstruction was 1–5 weeks. Six patients (3 females and three males) received SB ACLR and 14 patients (1 females and 13 males) received DB ACLR. All patients had returned to pre-injury activity by 12 months post-operative.

### Changes in bone mineral density over time

Before introducing vBMD value of each ROI for comparison and calculating change percent of vBMD, the reproducibility of each ROI mentioned above was examined. In terms of the ICC, the intra and interobserver reliabilities were all >0.9, which implied very good reproducibility (Table 1).

**TABLE 1:** Inter- and intraobserver reliabilities to determine the ROI.

ROI	Interobserver reliability, ICC (1.1)					Intraobserver reliability, ICC (2.1)				
	Preoperation	Postoperative 1M	Postoperative 3M	Postoperative 6M	Postoperative 12M	Preoperation	Postoperative 1M	Postoperative 3M	Postoperative 6M	Postoperative 12M
AM	0.960	0.941	0.947	0.943	0.958	0.971	0.968	0.987	0.980	0.967
PM	0.956	0.949	0.956	0.931	0.955	0.980	0.984	0.965	0.976	0.987
AL	0.947	0.950	0.944	0.945	0.940	0.976	0.968	0.987	0.984	0.965
PL	0.941	0.948	0.958	0.949	0.947	0.978	0.945	0.981	0.975	0.967

**FIGURE 3:**
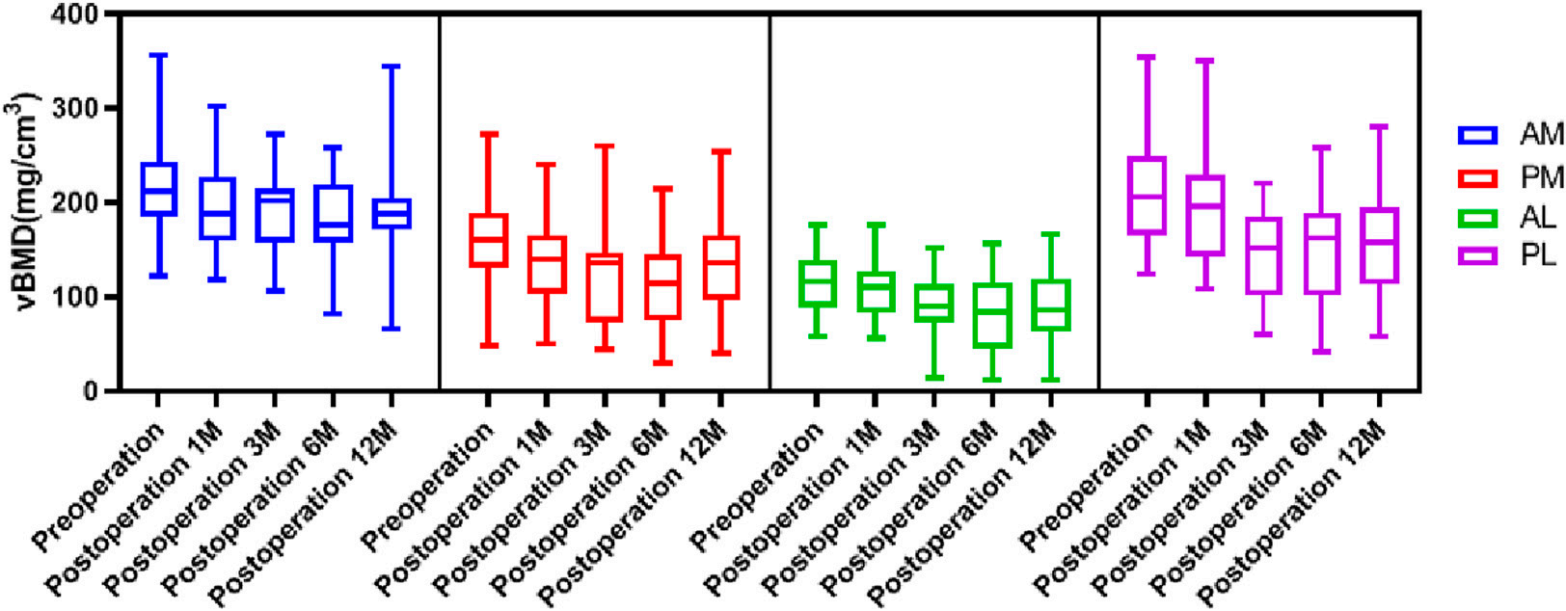
The distribution of vBMD over time at each ROI.

The general distribution vBMD in the tibial plateau was consistent at all timepoints. The AM region had the highest value of vBMD, followed by the PL region, and the vBMD was lowest at the AL region (Figure 3). There were significant differences in vBMD between the ROIs at the same time point, except for in the AM and PL regions preoperative and 1 month postoperative (Table 2). The fluctuation in vBMD over time was also similar in specific regions at each time point, with the value decreasing up to 6 months postoperative, before increasing again up to 12 months (Figure 3). However, the change patten was different between regions. Significant vBMD decrease can be found from postoperative 1 month at the AM, but from postoperative 3 months at the AL and PL regions. Then there was no significant difference between the subsequent timepoints. At the PM region, significant differences can be found between the preoperative and the other timepoints and between 6 months postoperative and the other timepoints (Table 2).

**TABLE 2:** The vBMD of each ROI at each timepoint.

vBMD (mg/cm^3^)	Preoperation	Postoperative 1M	Postoperative 3M	Postoperative 6M	Postoperative 12M	SNK-q test between timepoints at same ROI
AM	220.39 ± 57.21^a^	197.49 ± 49.66^b^	188.00 ± 44.85^b^	182.87 ± 42.08^b^	192.09 ± 46.69^b^	*p* < 0.05 between superscripts with none same letter, but >0.05 between superscript with a same letter
PM	162.40 ± 54.97^a^	139.57 ± 46.77^b^	122.41 ± 48.7^bc^	115.34 ± 45.98^c^	133.34 ± 49.89^bc^	
AL	116.05 ± 31.17^a^	110.25 ± 35.62^a^	91.44 ± 35.91^b^	84.14 ± 41.39^b^	89.69 ± 39.59^b^	
PL	207.93 ± 55.06^a^	196.42 ± 60.02^a^	147.00 ± 46.67^b^	152.40 ± 59.47^b^	156.00 ± 53.85^b^	
SNK-q test between ROIs at same timepoint	*p* < 0.05 except between AM and PL	*p* < 0.05 except between AM and PL	*p* < 0.05	*p* < 0.05	*p* < 0.05	

M, month.

**FIGURE 4:**
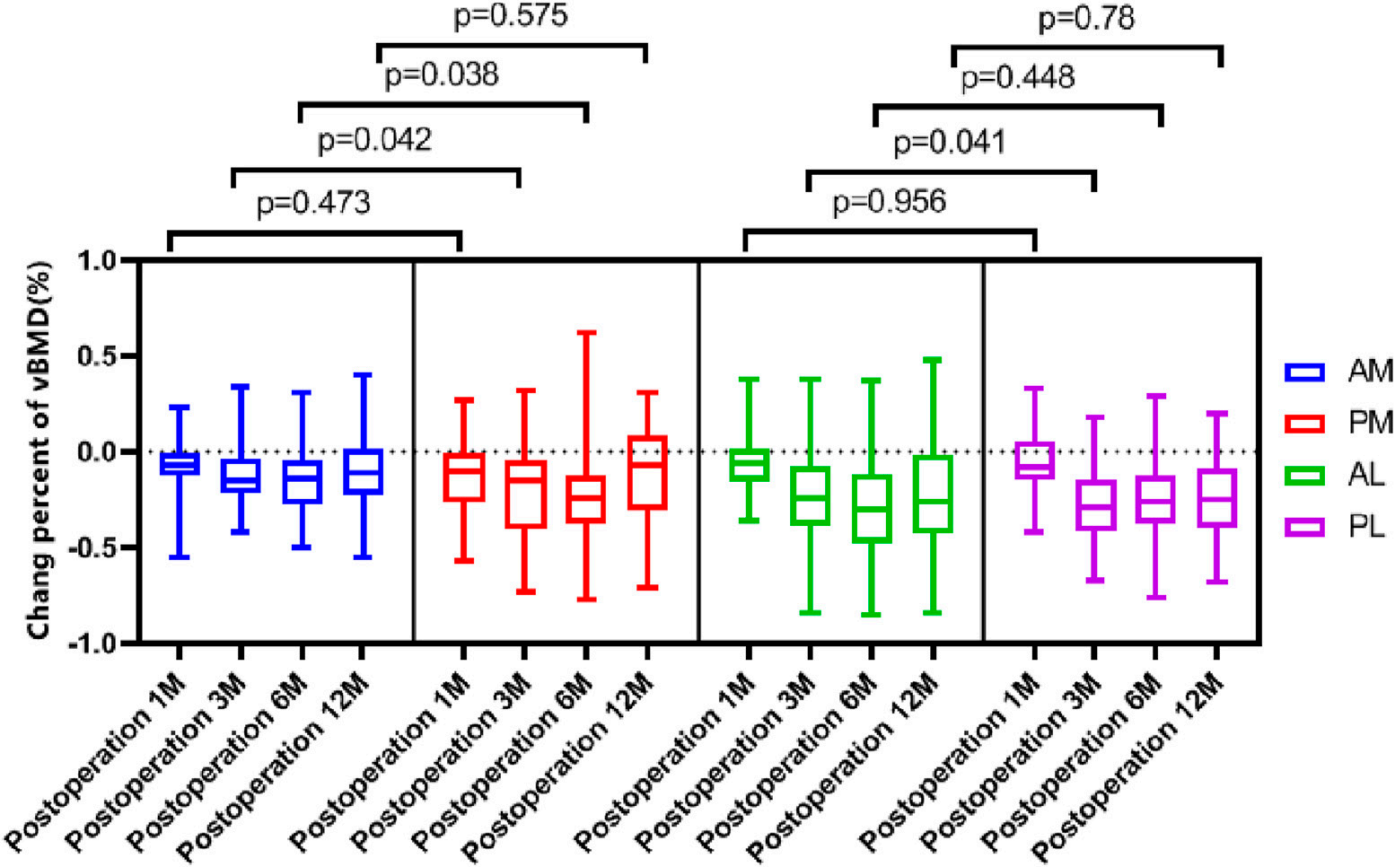
The change percent of vBMD over time at each ROI.

**FIGURE 5:**
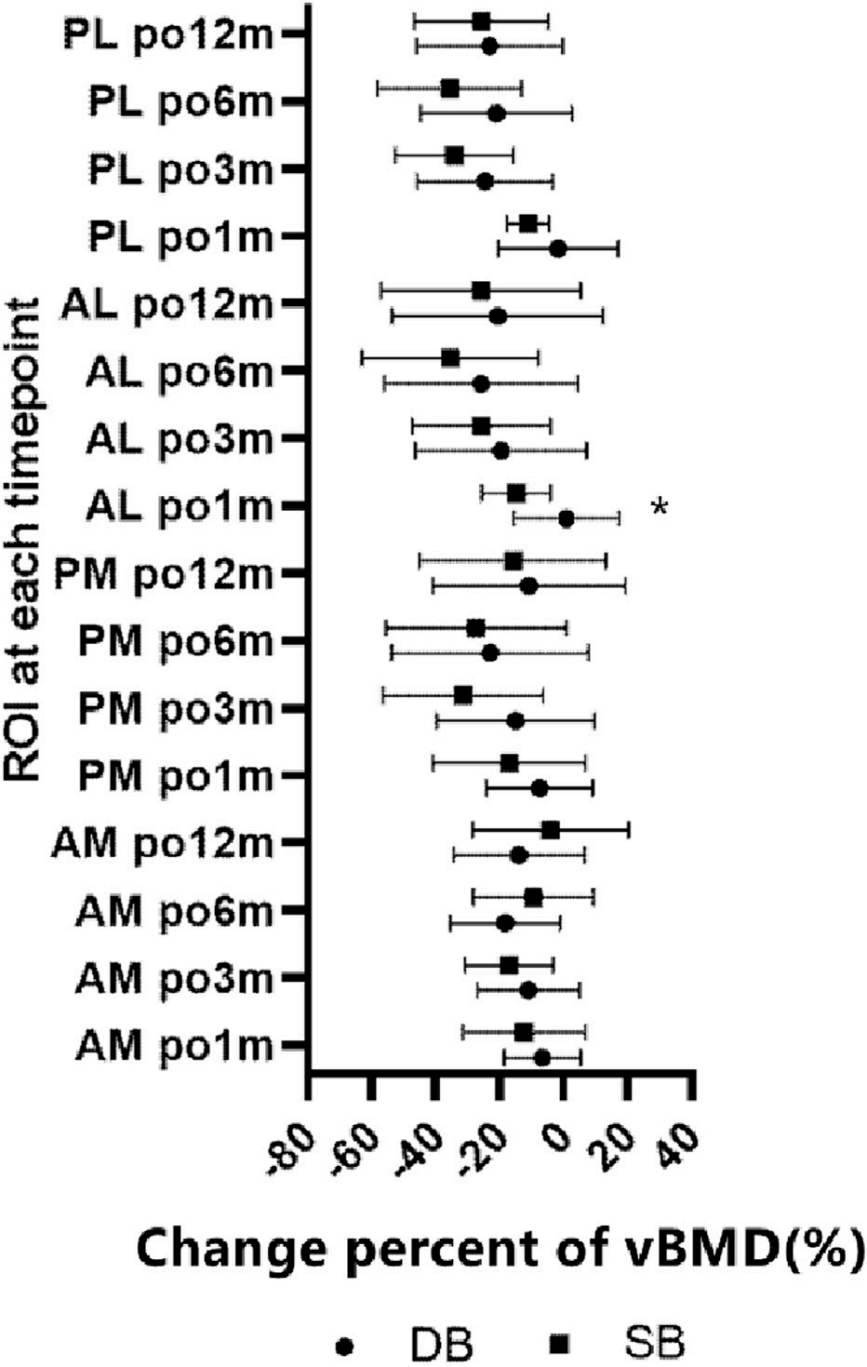
The change percent of vBMD between SB and DB ACLR. The squares and the dots indicate the mean values, and asterisk indicates a statistically significant difference between SB and DB groups at 1 month postoperative in the AL region.

The change percent of vBMD at the AM region were all comparable at each timepoints, and all comparable except postoperative 1 month at AL and PL region (Table 3). When comparing the change percent between AM and PM regions, it was comparable at postoperative 1 and 12 months, but was significantly greater at the PM region at 3 and 6 months. Whereas the change percents between AL and PL regions were similar at each timepoint except for at 3 months, with a greater loss at the PL region (Figure 4). When comparing the change percent of vBMD between DB ACLR and SB ACLR, there were no statistical differences at each ROI at 1, 3, 6, and 12 months postoperatively, except for the at AL region at postoperative 1 month (Figure 5).

**TABLE 3:** The change percent of vBMD of each ROI at each timepoint.

Change rate of vBMD (%)	Postoperative 1M	Postoperative 3M	Postoperative 6M	Postoperative 12M	SNK-q test between timepoints at same ROI
AM	−8.77 ± 15.06^a^	−13.29 ± 15.18^a^	−14.84 ± 17.96^a^	−10.07 ± 22.2^a^	*p* < 0.05 between superscripts with none same letter, but >0.05 between superscript with a same letter
PM	−11.09 ± 19.8^a^	−21.29 ± 25.57^bc^	−24.57 ± 29.3^c^	−12.66 ± 29.23^ab^	
AL	−5.11 ± 16.29^a^	−21.88 ± 24.67^b^	−29.49 ± 29.14^b^	−22.57 ± 31.77^b^	
PL	−5.26 ± 15.76^a^	−28.24 ± 20.42^b^	−26.62 ± 23.96^b^	−24.08 ± 21.69^b^	

M, month.

### *Post-hoc* analysis

*Post-hoc* analyses showed a power of at least 0.95 to support the significant differences of vBMD between the AM and PL regions (AM > PL) at 3 and 12 months, and that between the PL and PM regions (PL > PM) except for 3 and 12 months, as well as that between the PM and AL regions (PM > AL) at pre-operation and 12 months after surgery. The power was 0.95 for supporting the significant increase of vBMD at 6 months compared with the value at pre-operation, except for the AL region, where the power was shown to be 0.69. The power was less than 0.6 for supporting the significant decrease of vBMD at 12 months post-operative comparing with that at 6 months post-operative. The powers for supporting the significant larger change rate of vBMD at PM region comparing with the AM region were 0.52 and 0.54 respectively for the time points of 3 and 6 months post-operative. The power was 0.53 for supporting the significant larger change percent of vBMD at PL region when compared with the AL region at 3 months post-operative. The power was 0.73 to support a significant difference of change percent of vBMD comparing the DB and SB techniques at the AL region 1 month post-operative.

## Discussion

The present study revealed how ACLR affects the vBMD of trabecular bone over time in different regions of the tibial plateau of the operated leg during 1-year follow up. Previous studies have shown that ACL injury can lead to bone loss regardless whether the injury was treated through ACLR, possibly because of reduced weight bearing, prolonged disuse, or immobilization (Zerahn et al., 2006). The reported affected sites include the proximal tibia, proximal and distal femur, patella, hip region and calcaneus, and potentially may involve the entire leg (Stener et al., 2013). This disuse induced osteoporosis can be explained by Wolff’s law and Frost’s “mechanostat,” whereby bone growth and bone loss are stimulated by the local mechanical environment of the bone (Frost, 1997). These bone growth and loss manifested as variations of bone mineral content and microstructure, which can be well detected with pQCT as change of vBMD. Trabecular bone in the tibial plateau is subject to greater change than cortical bone, which can be more easily detected by pQCT (Mundermann et al., 2015). Subchondral bone is a dynamic tissue that is constantly remodeling, being continuously formed by osteoblasts and resorbed by osteoclasts (Chu et al., 2019). Compared with articular cartilage, subchondral bone has a relatively greater stiffness and strength, and absorbs most of the biomechanical forces transmitted from the articular surface (Zhen and Cao, 2014). It also undergoes modeling and remodeling more rapidly than cartilage with changes in the biomechanical environment (Goldring, 2012). Dynamic loading is known to increase the production of new bone, and has been shown to increase bone density following ovariectomy-induced osteoporosis in mice (Li et al., 2013). And bone formation is highly dependent on the amplitude and frequency of loading (Robling et al., 2002). This current study similarly found that the vBMD at all regions decreased up to 6 months postoperative, and then steadily began to increase again. In general, this follows the postoperative rehabilitation process, where partial weight-bearing and full weight-bearing are only allowed at postoperative 2 and 3 months respectively. The delay from weight-bearing (at 2 or 3 months) to reversing the loss of bone stock (6 months) may be due to the gradual stimulation of bone formation. It has also been reported that initial bone loss can occur during accelerated rehabilitation (Evans et al., 2012; Nyland et al., 2010), and the vBMD may not returned to normal levels even after 24 months (Zerahn et al., 2006).

However, the change in vBMD in this study was not the same in all regions, which may result from the unevenly distributed articular stress which is related to the knee kinematics. Normal knee motion requires posterior translation of the femur and external axial rotation of the tibia during knee flexion (Johal et al., 2005). Rupture of the ACL or poor functional performance of an ACL graft after ACLR can lead to considerable changes in knee kinematics, including translational and rotational laxity and/or offset (Asmakutlu et al., 2021; Patterson et al., 2020; Takeda et al., 2014). Furthermore, the aberrant kinematics can alter the stress distribution in the joint and cause post-traumatic osteoarthritis (Kvist et al., 2020). Theoretically, after the decline in vBMD following immobilization, the recovery of vBMD in each region should correspond to the stress experienced during loading. Differences in the loss and recovery of vBMD between the regions may indicate a change in the loading pattern on the tibial plate. Bo Zerahn et al. measured the vBMD of two 1 cm^2^ regions located 2 mm below the medial and lateral tibial subchondral plate after ACLR using dual-photon absorptiometry and found that bone loss was most pronounced on the lateral side (Zerahn et al., 2006). It is consistent with the finding of the current study, the bone loss and recovery were not universal throughout the subchondral bone of tibial plateau. However, it is limiting to only consider the tibial plateau as being divided into medial and lateral sides, without considering the anterior and posterior regions separately. The different regions come into play as the femur articulates on the tibial plateau during flexion (Bolcos et al., 2020). Therefore, four ROIs on the tibial plateau were identified in current study including AM, PM, AL and PL. The preoperative vBMD was found to be greatest in the AM region, followed by the PL region and PM region. The vBMD was lowest at the AL region, being almost half the value of the AM region. This is in accordance with normal knee kinematics. For the non-weight bearing knee kinematics, the lateral femoral condyle moved posteriorly 22 mm from knee joint hyperextension to 120° flexion. From 120° to full squatting there was another 10 mm of posterior translation, with the lateral femoral condyle appearing almost to sublux posteriorly. But the medial femoral condyle demonstrated only minimal posterior translation until 120° (Johal et al., 2005). The rare slide of the lateral femoral condyle to AL region of the tibial plateau results in the lowest vBMD, whereas the PL region possesses higher vBMD resulting from the posterior slide of the lateral femoral condyle. Retrieved unicompartmental tibial plateaus resected because of antero-medial osteoarthritis have shown a similar elliptical pattern of loss of cartilage in the antero-medial part of the tibial plateau, regardless of the health of the ACL (Rout et al., 2013). This phenomenon may be induced by the medial femoral condyle sliding anteriorly on the tibial plateau under weight-bearing, which may consequently stimulate bone formation at the AM region. For the knees with a deficient ACL or after ACLR, both the internal rotation and posterior sliding of the femoral condyle can increase above the level experienced in a healthy knee, and the abnormal kinematics may persist in the medium to long-term after ACLR (Asaeda et al., 2017; Hasegawa et al., 2015). The internal rotation may lead to the femoral condyle contact point moving posteriorly on the medial tibial plateau but anteriorly on the lateral tibial plateau. And the concurrent posterior sliding can force the contact point to move further posteriorly on the medial plateau, but also counteract the anterior move of the lateral condyle. This may be the reason why the vBMD at the PM region decreased more significantly than that at the AM region at 3 and 6 months postoperatively, but recovered more significantly after 12 months. And this phenomenon was not observed at the AL and PL regions. The posterior movement of the femoral condyle on the lateral side results in greater stress and a corresponding rise in vBMD, which is in agreement with studies reporting cartilage damage on the posterior side with the progress of ACL damage (Rout et al., 2013). However, this study did not find any significant difference between SB and DB ACLR, which may indicate that the resulting knee kinematics do not differ significantly between these two methods. Since the DB reconstruction technique was first proposed, it has been debated whether it is superior to SB reconstruction. Also, DB ACRL can present additional complications, such as convergence of the bone tunnels, difficulty in operating on small knees, and difficulty with recreating bone tunnels in cases of revision surgery (Harner and Poehling, 2004). A number of comparative studies have been performed to assess these two methods, but even the results of recent meta-analyses were inconclusive regarding knee stability, clinical function and OA changes (Chen et al., 2018; Dong et al., 2019; Mascarenhas et al., 2015). The results of this current study are consistent with recent findings of Fu et al., in which study no significant differences were found between SB and DB ACLR in terms of primary kinematic variables, and both reconstruction techniques were effective at restoring near-normal dynamic knee function (Tashman et al., 2021).

## Limitations

This study has limitations. First, there was no control group of healthy knees or a group with ACL deficiency. These groups were not considered because of the clinical risk with performing repeated CT examinations on healthy volunteers. And ACL deficiency with non-surgical treatment may obviously change the knee kinematics and gait from which after ACLR and may induce osteoarthritis quickly. Second, the sample size was limited, with only 20 patients completing the last follow-up. This is due to high rate of patients lost to follow-up, mainly because of concerns about the detrimental effect of CT scanning, and partially because of pregnancy or personnel transfer of the subjects. Third, patients with ACL rupture often concomitantly suffer from meniscus tear, but the effect of menisectomy or meniscal repair on subchondral vBMD cannot be eliminated. It has also been found that menisectomy is a strong risk factor for developing osteoarthritis after ACLR (Barenius et al., 2014). This bias may be eliminated by recruiting patients with isolated ACL rupture but without meniscal tearing. In addition, only CTXA Hip and Volumetric Spine modules were available in the pQCT workstation, while there was no module for the knee. The Volumetric Spine module was used to measure vBMD in this study. Although the data may not be the true value of vBMD at the tibial plateau because the difference of normalization value between modules, these data can be used for comparison between different ROIs and timepoints. And determination of size and location ROI are subjective, two well-trained radiologists cooperated during the procedure, and keep constant on size and location ROI at each timepoint of the same patient as possible, which inevitably results in inaccuracy. Finally, the duration of follow-up was short-term at 12 months, but following up will be proceeded to observe the long-term outcomes on vBMD.

## Conclusion

In conclusion, the vBMD was not evenly distributed throughout regions of the subchondral bone at the tibial plateau. A partially reversible decline in vBMD was found in all regions at 12 months after ACLR. But the change percent varied among the different regions, which may indicate inadequate restoration of knee kinematics after ACLR. DB and SB ACLR methods may have a similar effect on knee kinematics.

## Data Availability

The raw data supporting the conclusions of this article will be made available by the authors, without undue reservation.

## Abbreviations

ACL, Anterior cruciate ligament; ACLR, Anterior cruciate ligament reconstruction; SB, Single-Bundle; AM, Anteromedial; DB, Double-bundle; BMD, Bone mineral density; pQCT, Peripheral quantitative computed tomography; vBMD, Volumetric bone mineral density; ROM, Range of motion; ROI, Regions of interest; PM, Posteromedial; AL, Anterolateral; PL, Posterolateral; ICC, Intraclass correlation coefficients.
